# A Massive Open Online Course for Primary Prevention of Stroke: Evaluation of Co-Design Process

**DOI:** 10.2196/89358

**Published:** 2026-10-20

**Authors:** Aastha Gurung, Seamus Barker, Sabah Rehman, Rachel E Climie, Rosanne Freak-Poli, Catherine Burns, Monique F Kilkenny, Tara Purvis, Christine Farmer, Brenda Booth, Janet E Bray, Dominique A Cadilhac, Jan Cameron, Lachlan L Dalli, Stephanie Ho, Eleanor Horton, Timothy Kleinig, Lisa Murphy, Mark R Nelson, Muideen T Olaiya, Amanda G Thrift, Seana L Gall

**Affiliations:** 1 Menzies Institute for Medical Research, University of Tasmania, 17 Liverpool St, Hobart, Tasmania, 7000, Australia, 61 3-6226-4728; 2 Tasmanian School of Medicine, University of Tasmania, Hobart, Tasmania, Australia; 3 Stroke and Ageing Research, Department of Medicine, School of Clinical Sciences, Monash University, Clayton, Victoria, Australia; 4 School of Public Health and Preventive Medicine, Monash University, Melbourne, Victoria, Australia; 5 Stroke and Critical Care Research Theme, Florey Institute of Neuroscience and Mental Health, University of Melbourne, Heidelberg, Victoria, Australia; 6 Stroke Foundation, Melbourne, VIC, Australia; 7 Adelaide Medical School, The University of Adelaide, Adelaide, South Australia, Australia

**Keywords:** stroke, cardiovascular disease, co-design, coproduction, cocreation, massive open online course, preventive medicine, MOOC

## Abstract

**Background:**

Public awareness and understanding of stroke are crucial to reducing its overall burden. Massive open online courses (MOOCs) are free, self-directed online learning platforms that provide accessible education.

**Objective:**

This study aimed to describe the process, outcomes, and evaluation of co-designing the Preventing Stroke MOOC.

**Methods:**

The co-design process was conducted with various stakeholder groups from July 2020 to December 2024 in Australia over three phases: (1) predesign, (2) co-design, and (3) postdesign. In the predesign phase, we consulted with MOOC researchers and held a half-day in-person workshop with community members to prioritize the stroke prevention topics, structure, format, and features, followed by user testing of a single developed module. The larger study comprised 16 virtual focus groups (14 co-design and 2 user testing focus groups) with community members and health knowledge experts. Of the 14 co-design focus groups, 12 informed the MOOC design, including content, structure, and delivery. This was followed by drafting content for each module, expert review (eg, clinicians and researchers), and development of platform materials. In the postdesign phase, we conducted user testing to collect feedback and evaluated the co-design process to determine how participants’ input shaped the final MOOC design.

**Results:**

In the predesign phase, 5 MOOC experts and community members contributed to a pilot module comprising 9 pages on the prioritized topics, including a focus on stories from people with lived experience of stroke. A total of 8 people tested this module, providing positive feedback about content and delivery. The co-design phase engaged 22 participants across 12 focus groups relevant to the MOOC. Their suggestions contributed to the multimedia design of the 7 core modules, with personalization via 9 optional mini-modules. Feedback on technical issues and suggested improvements identified during postdesign user testing were resolved. Evaluation of the MOOC co-design process found that the majority of participants’ suggestions gathered during the co-design phase were included in the final MOOC.

**Conclusions:**

A structured, co-design process resulted in the Preventing Stroke MOOC reflecting participants’ suggestions and provided important insights on the educational information needed to inform the general population about stroke prevention.

## Introduction

Stroke is largely preventable, with a set of common modifiable risk factors accounting for nearly 90% of the population-attributable risk of stroke [1]. Modifiable risk factors include physical inactivity, smoking, inadequate diet, and excessive alcohol consumption, as well as biomedical factors such as hypertension, atrial fibrillation, diabetes, obesity, and dyslipidemia [1,2]. Such factors are common, with almost all adults in Australia having at least 1 risk factor and nearly 88% having 2 or more risk factors for stroke [3]. Consequently, increasing public knowledge of risk factors for stroke is crucial for enhancing prevention efforts [4-6]. In Australia, the Stroke Foundation runs 2 educational campaigns aimed at improving the knowledge of stroke risk factors and warning signs in the community [7-9]. The first campaign involves mass media promotion of face, arms, speech, and time (FAST) stroke signs. The second campaign is the StrokeSafe Speaker program, which involves presentations to the community, usually delivered by volunteers with lived experience of stroke. While there has been an improvement in the understanding of stroke in the community since the first national campaign in 2004 [7-9], many Australians cannot identify stroke warning signs and fail to call emergency medical services when a stroke occurs [10]. This may be related to the incomplete retention of knowledge and limited behavior change from these types of educational interventions [6,7]. Kilkenny et al [7] demonstrated that the knowledge gained by attendees to the Stroke Foundation’s StrokeSafe Speaker program diminishes within 3 months posteducation. An important consideration in educating the community about stroke is that interventions should achieve sustained knowledge and actions toward stroke prevention.

The global growth of internet use has created opportunities to provide accessible health information, interventions, and services [11]. Massive open online courses (MOOCs) are free and flexible web-based learning tools [12,13]. MOOCs enable the delivery of education to a large audience through video lectures, quizzes, additional resources, and discussion boards [12]. Numerous MOOCs are available covering a wide range of health promotion topics [14]. A search of major online learning platforms, including MOOC List, edX, and Coursera, using the keyword “stroke” identified courses that covered topics such as stroke anatomy, recovery, and endovascular treatment patient selection, but none specifically addressed stroke prevention. To the best of our knowledge, there are currently no MOOCs focused on primary stroke prevention. Such a MOOC has the potential to educate the general public with evidence-based information about stroke, increase awareness of prevention, support recognition of individual risk, and promote positive behavior change.

Participatory research approaches, such as co-design, are widely used to develop inclusive health interventions [15]. Co-design is an iterative process involving active and meaningful collaboration between stakeholders with diverse expertise throughout the design process [16], with the aim of tailoring the intervention to the needs of the intended audience [15-17]. Co-design has been increasingly used to develop effective and sustainable health interventions [18]. For MOOCs aimed at improving health-related knowledge and understanding, the quality of educational content plays a critical role in achieving learning outcomes [19,20]. Co-designing MOOCs with stakeholders, informed by real-world needs and experiences, can enhance content relevance for intended users [19].

While co-design approaches are widely used in developing health interventions, limited emphasis has been placed on evaluating the co-design process [21]. Evaluating the co-design process is essential to determine whether the contributions of co-design participants, as active partners, were carried through to the end product, to strengthen understanding of how the process influenced intervention development, and to identify areas for improvement in future co-design activities [21].

Our aim was to undertake a co-design approach to develop a MOOC for primary stroke prevention to address poor knowledge and improve the identification and management of stroke risk factors in the community. Specifically, we aimed to:

- Describe the process and outcomes of co-designing the Preventing Stroke MOOC.
- Evaluate the co-design process of the Preventing Stroke MOOC to determine the extent to which participants’ suggestions from the co-design sessions were included in the final MOOC design.

## Methods

### Study Design

This study is part of a larger research project named “Love Your Brain: A digital health intervention to prevent stroke,” comprising two components: (1) a MOOC and (2) a text messaging system [22]. The co-design method [23], overall co-design results across both components of the platform [24], and the development of the text message system [25] have been described previously. This study used a qualitative research approach to co-design the MOOC and retrospectively evaluate the co-design process. Methods and results are reported according to the Consolidated criteria for Reporting Qualitative research (COREQ) checklist (Checklist 1) [26].

### Overview of Co-Design Process

The co-design of the Preventing Stroke MOOC was carried out in three iterative phases involving (1) predesign, (2) co-design, and (3) postdesign (Figure 1), broadly aligned with the generative co-design framework by Bird et al [27]. The co-design process occurred between July 2020 and December 2024. Data collection included consultation meetings, workshops, and focus groups, which were audio and video recorded where appropriate and supplemented by field notes. Participants in the co-design process comprised diverse stakeholder groups, including community members (with or without a history of stroke) and health knowledge experts (eg, allied health clinicians, nurses, and researchers). Throughout the process, participants were active partners, positioned as experts of their lived experience.

**Figure 1.**
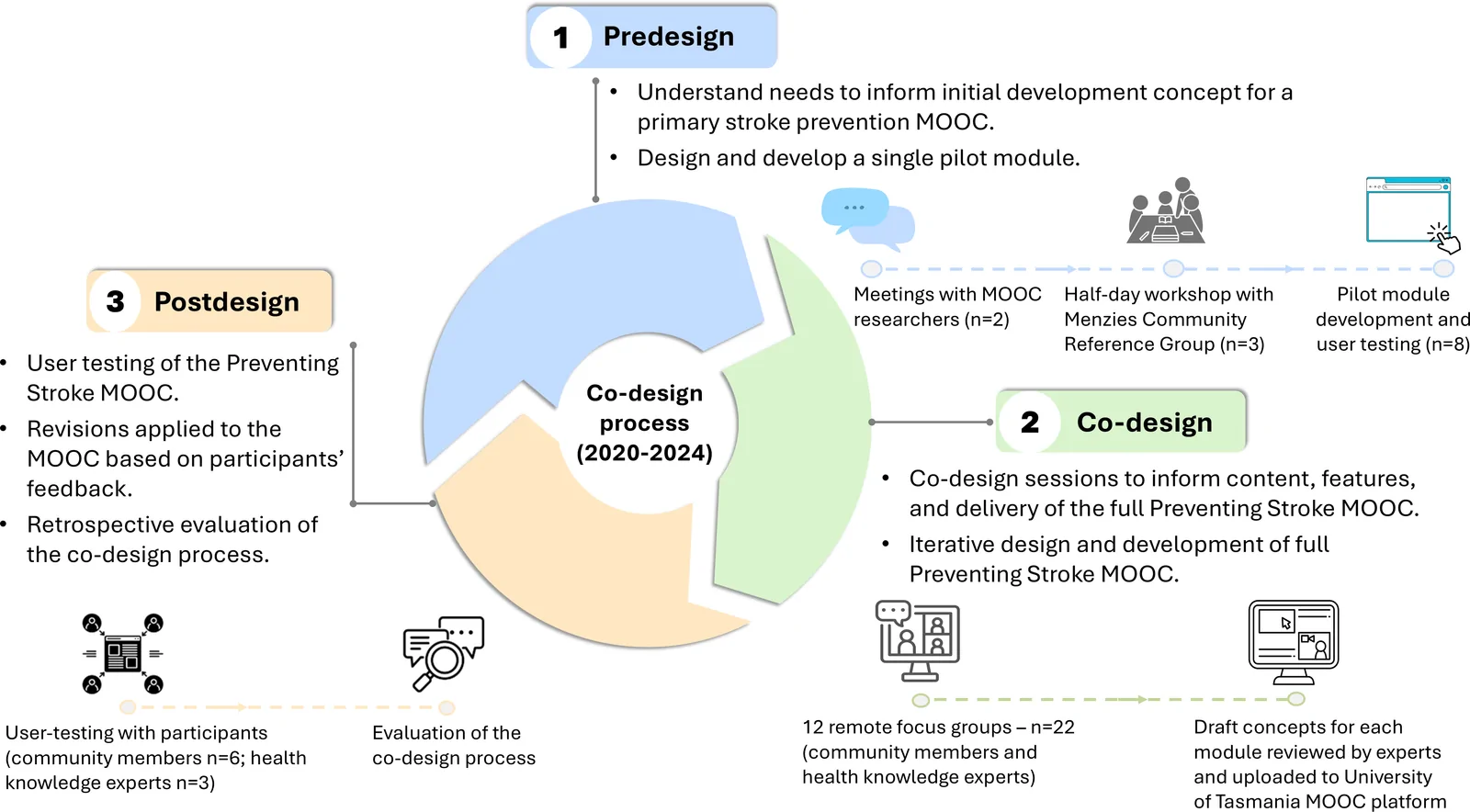
Co-design process. MOOC: massive open online course.

### Phase 1: Predesign (July 2020-March 2022)

#### Collecting Information to Understand Needs

The first stage of the predesign phase involved consultation meetings with researchers at the University of Tasmania (UTAS) who have experience in MOOC design and development. The aim was to understand the requirements related to the initial development concept for a stroke prevention MOOC. These researchers were emailed an invitation to participate in informal in-person consultation meetings on July 20 and August 13, 2020. The purpose of these meetings was to gain insights into their experience in designing health MOOCs for the community.

We then explored community perspectives on how a MOOC could support primary stroke prevention. Participants were recruited via convenience sampling of Menzies Institute for Medical Research (MIMR) Cardiovascular Research Flagship Community Reference Group (CRG). CRG members were invited via email to participate in an in-person half-day workshop at MIMR in March 2022. The workshop was facilitated by the MIMR Cardiovascular Research Flagship lead (SLG, female, PhD), a cardiovascular epidemiologist with experience in conducting qualitative research. The workshop focused on the perceived usefulness of a stroke prevention MOOC, topics for inclusion, preferred delivery modes, and strategies to engage learners. The Cardiovascular Research Flagship program manager (CF, female, Master of Environment [MEnv]) synthesized findings from this stage to consolidate insights from the meeting and workshop, guiding the initial design concept for the MOOC. This involved reading and interpreting the field notes from both sessions.

#### Development and User Testing of the Pilot Module

Based on the needs identified during the first stage, we developed a single pilot module to present the initial concept for the proposed Preventing Stroke MOOC. The module was drafted using existing evidence and resources, with content modeled from the Stroke Foundation’s StrokeSafe Speaker program.

Materials for the module included videos featuring experts (eg, epidemiologists and cardiologists) and people with lived experience (eg, survivors of stroke), who were invited to share their insights and stories. Experts from the research team’s network were invited to participate in video recordings, where they discussed stroke and risk factors from clinical, research, and public health perspectives. Invitations were sent to CRG members with lived experience of stroke to participate in video recordings to share their experiences of living with and managing stroke. Each video participant drafted their own script, which the research team then reviewed and refined together with them as needed. The filming took place in person in Hobart, Tasmania. The final draft of the module and video transcripts were reviewed by other members of the local research team and refined through iterative revisions until consensus was reached. The pilot module was developed in collaboration with the UTAS Digital Futures team, who supported page design, video uploads, and graphics integration. The pilot module was uploaded to the UTAS MOOC platform with restricted access.

Snowballing methods were used to recruit participants for user testing. Email invitations were circulated via the Stroke Foundation Lived Experience Council (formerly the Consumer Council). The Lived Experience Council is a board subcommittee within Stroke Foundation that provides insights into strategy and operations. Participants who agreed to take part were emailed a link to access the module. While working through the module, they were asked to reflect on the usefulness of topics, aspects they found engaging, any technical difficulties, and suggestions for informing future modules. To obtain feedback on the pilot module, we conducted 3 focus groups between March 3 and 23, 2022. The focus groups followed a semistructured guide (Multimedia Appendix 1). All sessions were facilitated by the flagship program manager (CF) and attended by the flagship lead (SLG), who took notes and responded to participant queries as needed. One researcher (AG) reviewed focus group transcripts and field notes to summarize participants’ feedback on the pilot module.

### Phase 2: Co-Design (April 2023-February 2024)

#### Data Collection Through Co-Design Focus Groups

The Preventing Stroke MOOC was part of the Love Your Brain project, which also involved co-designing a text message system [24]. Participants included two separate groups: (1) community members and (2) health knowledge experts involved in health education or promotion. Recruitment occurred between April and May 2023. Community members were approached using recruitment flyers distributed by email to consumer reference groups (eg, MIMR CRG and Stroke Foundation) and newsletters (eg, Heart Foundation consumer newsletter). Health knowledge experts were recruited through emails circulated within the research team’s professional networks, including research collaborators and clinicians (eg, nurses, stroke neurologists, physiotherapists, and occupational therapists). Focus group methods, including facilitation approach, discussion guide, and analysis, are described in detail elsewhere and are freely accessible [24]. A total of 16 focus groups were conducted as part of the larger study (14 co-design focus groups and 2 user-testing focus groups), of which 12 co-design focus groups were relevant to the MOOC and are reported in this phase [24]. In brief, separate sessions were held remotely via Zoom (Zoom Communications, Inc) for each participant group to mitigate power imbalances. Discussion topics included prompt questions framed around primary stroke prevention (see Multimedia Appendix 2 for the question list used in each focus group). Over 4 months (June-September 2023), participants worked together with the research team to inform design decisions for the MOOC, including content, level of detail, presentation format, delivery modes, and engagement strategies [24].

#### Development of Preventing Stroke MOOC

Findings from the co-design focus groups, existing guidelines on primary stroke prevention, and evidence from the literature informed the development of the MOOC. Video materials were developed for the modules, incorporating lived experience stories and insights from subject area experts. Some community members who participated in the co-design focus groups were invited to take part in filming. Subject area experts (eg, epidemiologists, cardiologists, neurologists, and paramedics) were recruited through the research team’s professional networks. Video scripts were drafted by speakers and reviewed by the research team before final review and approval by the speaker. Overall, 2 types of video styles were used based on findings from the co-design process, including direct-to-camera recordings and hand-drawn animations. Direct-to-camera videos featured speakers delivering short, scripted segments on topics relevant to their expertise. Animated videos were produced in collaboration with Russ Animates Ltd. A transcript of each video was available to download as a PDF to ensure accessibility.

Interactive features, including gamified activities such as flashcards and crosswords, were developed to ensure the content was engaging. End-of-module quizzes were created for each module, and links to additional resources were selected by the research team and subject area experts to complement the topics. Once the content and materials were finalized, they underwent iterative review and refinement in collaboration with subject area experts. The Digital Futures team of education technologists at UTAS supported the design of the MOOC platform. The content was uploaded to the MOOC page hosted by UTAS with restricted access. The entire development process took place over a 9-month period (May 2023-February 2024).

### Phase 3: Postdesign (March 2024-December 2024)

#### Preventing Stroke MOOC User Testing Data Collection

Invitations to participate in user testing of the MOOC were sent to the same participants who took part in the focus groups during the co-design phase. Additional participants were invited through the research team’s professional networks to provide broader perspectives. Participants who agreed to participate in user testing received a welcome email containing a link to access the MOOC and were given 8 weeks to engage with the course.

Feedback was collected through separate focus groups with the community and health expert groups remotely via Zoom in March 2024. The data collection method followed the same process as that used for the co-design focus groups [24]. The sessions focused on exploring users’ experiences with the MOOC, including overall perceptions of the course, features they found useful, challenges encountered, and suggestions for improvement. In addition, 3 health knowledge experts who had not participated in the co-design phase were provided access to the MOOC and asked to review the course and provide written feedback via email regarding content, usability, and suggestions for improvement. The research team reviewed the feedback and identified areas for improvement. These were discussed in team meetings and shared with the UTAS Digital Futures team, with revisions made to the MOOC where feasible, technically possible, and within budget.

#### Preventing Stroke MOOC User Testing Data Analysis

Transcripts from the user testing focus group were analyzed manually in Microsoft Word. One researcher (AG) reviewed and summarized participants’ feedback using a deductive approach [28]. Feedback from the community member focus group and email feedback from health knowledge experts were organized according to the main categories of the user-testing discussion guide (eg, general impressions, useful features, challenges encountered, and suggestions for improvement) and grouped under a single overarching theme reflecting overall feedback on the MOOC. The process involved frequent discussion with one of the focus group facilitators (SB, male, Research Fellow, PhD), and findings were reviewed and confirmed by the research team.

#### Evaluation of the Co-Design Process

We conducted qualitative content analysis to retrospectively evaluate the co-design process and determine whether participants’ input from the co-design phase was incorporated into the final MOOC design [28,29]. The analysis followed 3 steps.

In the first step, one researcher (AG) engaged with the MOOC platform as a mock participant and worked through each module. The MOOC was treated as qualitative data, and all module content was reviewed in detail. As each module was completed, key design features of the course were identified and described (eg, whether learning objectives were present, the structure of modules, topics covered, the number and type of learning activities, and the media formats used). These descriptions were used to characterize how the MOOC was designed. Word counts were calculated for each module, including video transcripts, using Microsoft Word, and readability was assessed using the Flesch Reading Ease score [30].

In the second step, 12 transcripts from co-design focus groups involving 22 participants were imported into the NVivo software (version 15; Lumivero). The analysis began with a close reading of the transcripts. Meaning units were identified inductively as words, sentences, or short passages conveying a single idea related to the MOOC [28,29]. These meaning units were then interpreted to understand participants’ suggestions in relation to the MOOC design. An initial subset of transcripts was analyzed first to explore how participants’ suggestions were being interpreted. These interpretations were discussed with 2 researchers (SB and SLG), who were involved in the co-design facilitation, to ensure the analysis was credible and meaningful within the context of the co-design process. The remaining transcripts were then analyzed, with ongoing discussion with SB and SLG. This involved revisiting selected excerpts, considering alternative interpretations, and refining the understanding of participants’ suggestions.

In the final step, we undertook triangulation by comparing findings from the MOOC and the co-design focus groups to examine whether and how participants’ suggestions were reflected in the final MOOC design [31]. To support this comparison, we drew on design elements adapted from the Analysis, Design, Development, Implementation, and Evaluation (ADDIE) instructional design model [32], which outlines key aspects of course design, comprising learning objectives, structure, content, exercise, assessment, and media selection. Features identified in the MOOC were organized in relation to these design elements (Multimedia Appendix 3), and participants’ suggestions were subsequently examined alongside corresponding MOOC features for each design element. The number of meaning units identified in the second step was summarized descriptively by design element during this process to support transparency in reporting the distribution of participants’ suggestions. An example of the analytic process is provided in Multimedia Appendix 4.

Trustworthiness of the analysis was supported through strategies aligned with criteria for qualitative content analysis, including credibility, dependability, and confirmability [33]. Credibility was supported through ongoing discussions of interpretations with researchers who were involved in the co-design facilitation and contributed to interpreting the data. Dependability was supported through clear documentation of the data analysis process. Confirmability was supported by revisiting the transcripts and discussing interpretations to ensure they were grounded in the data. The analysis was primarily conducted by a researcher (AG) who was not involved in the co-design process, which helped reduce the influence of prior assumptions when interpreting participants’ suggestions and how they were reflected in the MOOC. In this context, interrater reliability was not considered appropriate for the approach taken, as the aim was interpretive understanding, with interpretations discussed with the research team [34,35].

### Ethical Considerations

Ethical approval for this study was obtained from the UTAS Human Research Ethics Committee (#27108) and the Monash University Human Research Ethics Committee (#35899; Universal Trial Number U1111-1305-2964). Verbal informed consent was obtained from all participants prior to participation, including consent for audio and video recording. This approach to consent was reviewed and approved by the human research ethics committees of each institution. Participants were reimbursed Aus $50 (US $34.70) for their time. Reimbursement was provided via bank transfer or gift voucher, according to participant preference. Participants were assigned unique study identification numbers, and identifying information was removed from transcripts prior to analysis. Study data were stored securely and were accessible only to authorized members of the research team.

## Results

### Phase 1: Predesign (July 2020-March 2022)

#### Collecting Information to Understand Needs

Two researchers involved in the design and development of the Preventing Dementia MOOC and the Understanding Multiple Sclerosis MOOC attended separate consultation meetings with the research team. Together, they recommended the following to guide the development of a successful MOOC: (1) engage stakeholders early in the design process, (2) plan for development costs, and (3) provide feedback to learners.

A total of 3 CRG members participated in the half-day workshop. They emphasized the importance of personalizing content to make it relevant and engaging for learners. Key elements recommended for inclusion were personalized stroke risk assessments and interactive features such as quizzes and discussion boards. Participants highlighted that the MOOC content should follow a structure similar to the StrokeSafe Speaker program, feature stories from people with lived experience of stroke, and include multimodal delivery through a combination of videos, graphics, and text to accommodate different learning styles.

#### Development and User Testing of a Pilot Module

The pilot module comprised 9 pages and covered topics that broadly followed the StrokeSafe Speaker program, including an introduction to stroke, living with stroke, stroke as a medical emergency, stroke statistics, understanding stroke risk, and managing stroke risk. The module featured 4 videos, with 2 presented by experts (a cardiovascular epidemiologist and a general practitioner) and 2 by people with lived experience of stroke. Additional materials included an end-of-module quiz, a discussion board, links to further resources, and a glossary.

A total of 9 participants were recruited for user testing of the pilot module. One participant opted out due to COVID-19. Participants were from Tasmania, New South Wales, Victoria, and Western Australia. Overall, 2 focus groups were held remotely via Zoom (n=6 participants), and 1 focus group was conducted in person at MIMR (n=2 participants). Each session lasted approximately 30 to 60 minutes.

All participants reported using a laptop or desktop computer to complete the pilot module. Overall, feedback was positive regarding the structure of the content, the topics covered, and the materials included. Participants described the module as engaging and easy to follow, indicating that it could be a useful tool for the general public. They further expressed that videos from people with lived experience of stroke had a particularly strong impact. Participants suggested that the full MOOC should include subtitles in the videos to improve accessibility. They also suggested that additional optional mini-modules could be incorporated, such as dedicated sections covering aphasia and childhood stroke.

### Phase 2: Co-Design (April 2023-February 2024)

#### Co-Design Focus Groups

In total, 22 purposively selected participants participated in at least 1 focus group (n=12 community members and n=10 health knowledge experts). Among the community members participating, 58% (7/12) were female, 58% (7/12) were aged <65 years, 75% (9/12) were stroke survivors, and 25% (3/12) had no personal history of stroke. Health knowledge experts were predominantly female (8/10, 80%), aged >35 years (6/10, 60%), and comprised equal proportions of researchers (5/10, 50%) and clinicians (5/10, 50%). Comprehensive thematic analysis of the co-design focus groups can be found in our previous publication [24]. In summary, specific to the MOOC, participants in the focus groups suggested including topics on stroke signs, risk factors, and the impact of stroke [24]. They emphasized ensuring the accuracy and credibility of information, the use of plain language, multimedia delivery formats, personalization through self-paced modules and topic choice based on learners’ risk factors, incorporating interactive elements, stories from people with lived experience of stroke, accessibility across major devices, and inclusive design [24]. They also specifically mentioned the benefit in using well-recognized evidence-based resources, including topics from the StrokeSafe Speaker program, to guide the structure of the MOOC content [24].

#### Development of Preventing Stroke MOOC

In total, 16 modules were designed, with 7 core modules and 9 optional mini-modules (Figure 2). The seven core content modules were (1) Introduction, (2) What is stroke, (3) Stroke numbers, (4) Stroke signs, (5) Risk factors for stroke, (6) Action plan, and (7) Completion. Optional mini-modules were designed to support personalization. These included 5 mini-modules focused on biomedical risk factors (blood pressure, cholesterol, atrial fibrillation, overweight and obesity, and blood sugar) and 4 mini-modules focused on lifestyle-related risk factors (smoking, diet and alcohol, exercise, and sleep). Participants were encouraged to complete at least 1 mini-module from each section. Participants received a certificate in the final module once they completed the course. A total of 60 individual videos were filmed with subject area experts (n=13) and individuals with lived experience of stroke or carers (n=8). We also developed 6 interactive activities such as flashcards and crosswords, 14 end-of-module quizzes, 110 graphics or infographics, and 129 hyperlinks to additional resources integrated into relevant modules.

**Figure 2.**
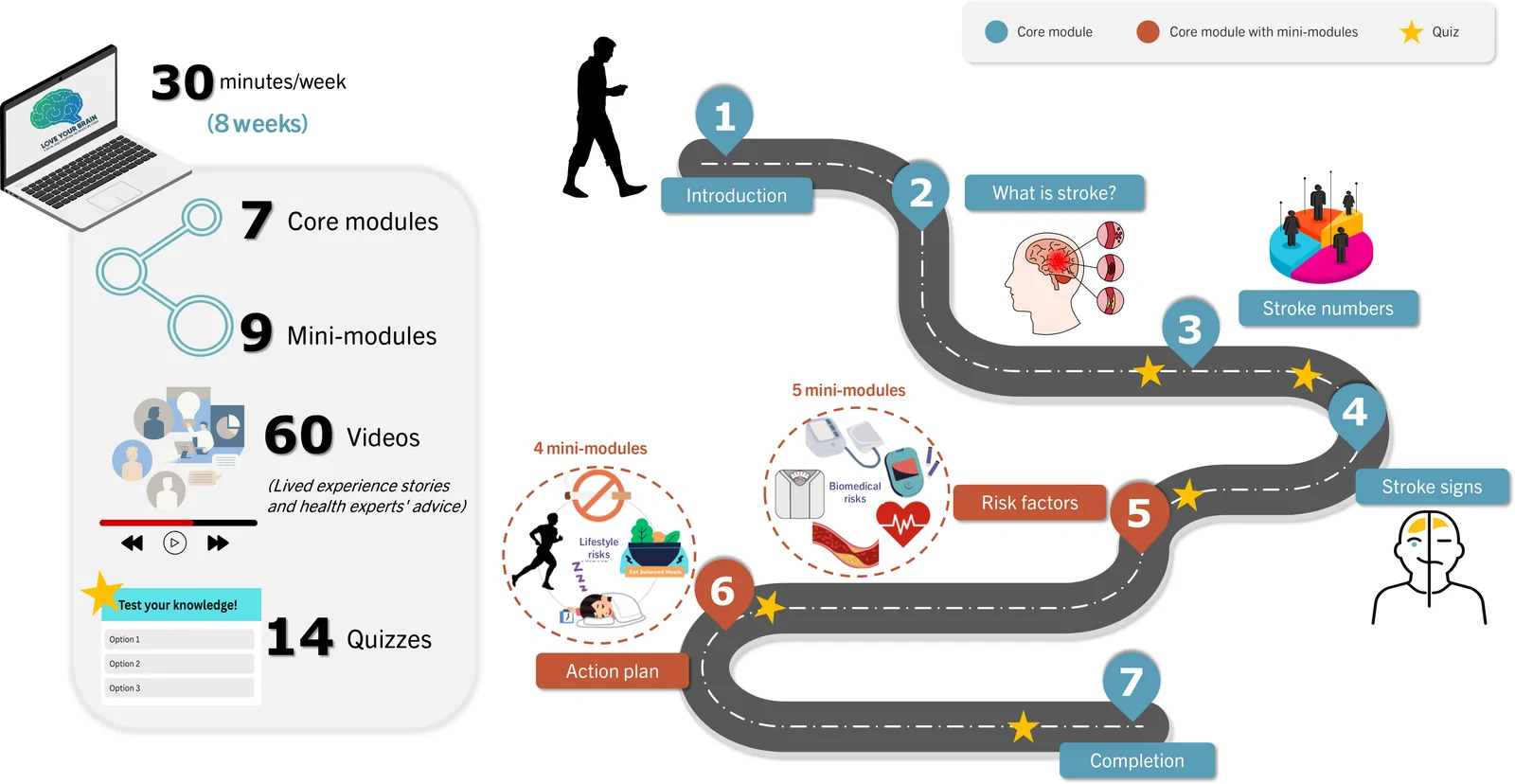
Preventing Stroke Massive Open Online Course learner journey.

### Phase 3: Postdesign (March 2024-December 2024)

#### Preventing Stroke MOOC User Testing

A total of 6 community members who had participated in the co-design focus groups undertook user testing of the MOOC. Health knowledge experts who participated in the co-design focus groups did not respond to the MOOC user testing invitation. Additional feedback was obtained via email from 3 health knowledge experts who had not been involved in the co-design phase. The focus group with community members was conducted online on March 13, 2024, and lasted approximately 60 minutes.

Overarching feedback from participants indicated that the MOOC was user-friendly, engaging, and well-structured. Participants also reported that the interactive features were useful and that the personalized risk assessment and videos provided impact. Participants noted a few technical issues, including difficulties accessing the MOOC via mobile phones and tablets.

Suggestions for improvement included defining key terms, maintaining consistent terminology throughout the content, and providing clear signposting, such as bolding keywords to support understanding and comprehension. Two participants noted that, from a disability perspective, graphics using similar colors may pose challenges for individuals with vision impairment or color blindness. They recommended high-contrast colors to improve visual accessibility. In collaboration with the Digital Futures team at UTAS, strategic modifications were made. These included technical updates to support compatibility across all major devices, refinement of wording for consistency, use of bold text for keywords, clear signposting, and use of high-contrast colors in graphics.

#### Evaluation of the Co-Design Process

Following review and documentation of the MOOC, the content covered a total of 81 topics, and multiple-choice questions (MCQs) were included in 14 modules (Multimedia Appendix 3). Data from co-design focus group transcripts (step 2) were analyzed to identify participants’ suggestions related to MOOC design, with 223 meaning units identified across the 12 transcripts. Triangulation of findings from step 1 and 2 involved examining how these suggestions were reflected in the MOOC. This comparison was conducted across the adapted design elements, with illustrative quotes presented in Tables 1–4. The findings are presented below, organized according to these elements.

**Table 1.** Participant suggestions from co-design focus groups (step 2) and comparison with MOOC^a^ design (step 3) for learning objectives and structure.

Design elements	Step 2: co-design focus groups			Step 3: triangulation	
	Participants’ suggestion	Meaning units	Example quotes	Suggestions integrated into MOOC	Observations
Learning objectives	Clear guidelines and expectations	2	“Having all those really clear set guidelines at the beginning would be an incentive, to know whether the people are going to sign up and keep going.” (Community member, P2)	Clearly stated learning objectives were present in all core modules, except for Module 7, which was a completion module and therefore did not require learning objectives.	Inconsistency in learning objectives across mini modules. 4 out of 9 mini modules did not state learning objectives
Structure	Layer knowledge across modules to build understanding Core modules for foundational learning, with optional modules on specific topics	11	“I thought it would be good to encapsulate all the information in a sequence…” (Community member, P3) “I really love the idea of having kind of like a sliding scale of the depth of information. So, you could have basic, moderate, and hardcore. Because I feel like even the general public sort of have this sliding scale of wanting information. Some people are happy with the basics, some people are thirsty for more.” (Health knowledge expert, P2)	Structure of almost all modules was linear. Module 5 and Module 6 core module which included mini modules had modular learning. structure	Suggestions were addressed.

a MOOC: massive open online course.

**Table 2.** Participant suggestions from co-design focus groups (step 2) and comparison with MOOC^a^ design (step 3) for content.

Design elements	Step 2: co-design focus groups			Step 3: triangulation	
	Participants’ suggestion	Meaning units	Example quotes	Suggestions integrated into MOOC	Observations
Content	Explain the word ‘stroke,’ use anatomical references Emphasize that stroke can affect people of all ages Include statistics Importance of recognizing the FAST^b^ signs of stroke Address modifiable and non-modifiable risk factors Visible and invisible impact of stroke Content should be simple and easy to understand Provide checklists and action plans for health appointments Include personal stories to enhance relatability	127	“Empowering of people to take those steps, to actually go to the doctor and to - I think prevention is so much about that empowerment and agency of people…” (Community member, P6) “It’s about they’re wanting to know what their individual risk is so rather than giving generic information, trying to make it as personalized as possible.” (Health knowledge expert, P4)	Module 2: builds foundational knowledge of brain anatomy and stroke types, explaining how strokes affect brain function. Module 3: explores childhood and perinatal stroke, stroke epidemiology, highlighting its prevalence, impact, and preventability. Features health experts’ videos and lived experience stories. Module 4: introduces the FAST test and other stroke warning signs, with multilingual resources and paramedic insights. Covers emergency response, diagnosis, treatment pathways, and telestroke services, supported by survivor stories. Module 5: covers modifiable and nonmodifiable stroke risk factors. Includes a personal risk assessment tool and mini-modules on biomedical risk factors for stroke. Module 6: covers what to expect during a stroke risk assessment appointment and includes mini-modules on lifestyle-related risk factors. Module 7: summarizes key learnings from all modules. Includes completion certificate.	MOOC content included all suggested topics, comprising 81 topics in total. One module had a readability score below 50, and 7 modules scored below 60. This poses challenges in accessibility and comprehension of these modules for people with low literacy levels.

a MOOC: massive open online course.

b FAST: face, arms, speech, and time.

**Table 3.** Participant suggestions from co-design focus groups (step 2) and comparison with MOOC^a^ design (step 3) for exercise and assessment.

	Step 2: co-design focus groups			Step 3: triangulation	
Design elements	Participants’ suggestion	Meaning units	Example quotes	Suggestions integrated into MOOC	Observations
Exercise	Include risk calculator or assessments Tailored feedback on exercises	7	“If we want to get people to the stage of changing behavior, they have to see themselves at risk and then make that appointment to find out the degree of risk that they might be at.” (Community member, P4) “…the risk calculator is really nice and personal, and it sort of filters people down that pathway to their risk.” (Health knowledge expert, P3)	Interactive activities were present in all core modules. Module 5 risk factors had cardiovascular health assessment with tailored feedback using traffic light system.	Suggestions were addressed.
Assessment	Include assessments at the end of the modules Avoid formal assessments	3	“If there’s activities at the end of each module that help people apply what they’ve learned, that may help reduce some of that frustration.” (Health knowledge expert, P3)	The MOOC did not include formal assessments. End of module quiz present at the end of all modules.	Suggestions were addressed.

a MOOC: massive open online course.

**Table 4.** Participant suggestions from co-design focus groups (step 2) and comparison with MOOC^a^ design (step 3) for media selection.

Design elements	Step 2: co-design focus groups			Step 3: triangulation	
	Participants’ suggestions	Meaning units	Example quotes	Suggestions integrated into MOOC	Observations
Media selection	Combine text, visuals, and videos to cater to different learning styles Include animated videos for clear understanding Videos with lived experience and health experts add credibility Further information with links to resources for users who want to learn in detail Multilingual options for learners from diverse linguistic backgrounds	73	“I really liked the graphics. I think showing the different areas of the brain and labeling how it can impact differently depending on which area the stroke attacks is really powerful and concise way to show it.” (Community member, P6) “…it is good to have a person with a professional in there [the videos], but I also think it is good to have somebody with lived experience.” (Health knowledge expert, P5) “I think it needs to be available in different languages and potentially links to information in language as well.” (Health knowledge expert, P2)	The MOOC included a mix of media including text, graphics, infographics, videos, transcripts, and hyperlinks to further information. Videos were short with an average length of 1 minute, 47 seconds.	Most suggestions were addressed. Multilingual options were not included.

a MOOC: massive open online course.

#### Learning Objectives

Two meaning units relating to learning objectives were identified. Participants emphasized the importance of setting clear expectations and guidelines at the beginning of the course.

*I think setting really clear guidelines at the beginning... Committing to something that’s going to run over weeks is a big task.*

[Community member, P1]

In the final MOOC, learning objectives were clearly stated on the landing page of all relevant core modules except Module 7, which served as a completion module and did not require learning objectives. Four of 9 mini-modules did not include learning objectives. This inconsistency may pose challenges in engagement and clarity for learners starting those modules.

#### Structure

There were 11 meaning units relating to the structure of the MOOC. Participants discussed having a logical sequence for the MOOC modules and topics. They expressed a preference for delivering content in a sequence of concepts beginning with foundational concepts and building upon them, followed by optional modules that learners can tailor depending on their perceived relevance.

*I was thinking that maybe the core content probably might be best to have that delivered in order for everybody… as people are going through them, the knowledge is layering up… Then when the individualized modules come up… they choose the module that they’re really the most interested in…*

[Health knowledge expert, P1]

The MOOC adopted a logical, linear structure across modules and within module topics. Core Module 5 on risk factors and Core Module 6 about action plans incorporated mini-modules that allowed learners to select topics relevant to them. The overall structure of the MOOC reflected the design discussed during the co-design sessions, supporting a logical learning structure and personalization.

#### Content

Content was most frequently discussed, with a total of 127 meaning units reflecting participants’ suggestions relating to content. Participants identified key content topics to include in the MOOC, such as types of stroke, statistics, signs and symptoms, and risk factors for stroke. They suggested contextualizing information to support comprehension and highlighting the relevance of the content to learners through personalization and lived experience stories. Participants also recommended presenting content in an aphasia-friendly format, using plain language, and aligning with health literacy principles.

*So, there’s a thing called universal precautions, which basically you assume everyone has low health literacy, and that way everyone can understand it; and part of that is using short, simple terms where you can..*

[Health knowledge expert, P1]

The MOOC incorporated content topics suggested by participants (Multimedia Appendix 3 for a list of content covered in the MOOC). Content across all modules was presented in simple language, supported by detailed explanations and examples provided through text and videos to foster understanding. Where technical terms were used in the content, they were bolded and detailed in a glossary section. The glossary consisted of 113 terms with simple definitions. The word count of modules ranged from 1688 to 4640 words, and readability scores ranged from 49 to 69 (equivalent to Australian Grade 8 to 12). The average readability score across modules was 60.7 (out of 100), with higher scores indicating easier readability. Seven of 16 modules scored below 60, with one mini-module (Cholesterol) scoring below 50, indicating that the content was relatively complex and may have included more technical language. Inconsistencies in readability scores may limit comprehension for learners with lower literacy. While an aphasia-friendly format or toggle was recommended, the MOOC did not include this feature.

#### Exercise

A total of 7 meaning units relating to exercises were identified. Participants suggested incorporating short and engaging interactive activities to enhance learner engagement.

*Having smaller bits throughout that are gamified or fun to do…something that’s less formal I think is going to be much more engaging for people, especially those with lower health literacy.*

[Health knowledge expert, P3]

Six interactive activities were included across all relevant core modules, including gamified crosswords, flashcards, and a cardiovascular risk assessment with tailored feedback. These features were not present in the mini-modules.

#### Assessment

Overall, 3 meaning units relating to assessment were identified. Rather than including formal assessments in the MOOC, participants preferred quizzes at the end of modules to allow learners to check their understanding without added pressure.

*I think having a more formal assessment at the end is going to be quite off-putting for people who don’t pass...*

[Health knowledge expert, P3]

The MOOC included 14 end-of-module quizzes across all relevant core modules and optional mini-modules, with an average of 3 questions per quiz.

#### Media Selection

A total of 73 meaning units relating to media selection were identified. Participants recommended using a diverse range of formats to deliver information. These included simple infographics, diagrams, short videos with minimal distractions, videos featuring both people with lived experience and health experts, and hyperlinks to additional resources.

*With a mix of the text as well and then somebody talking, so you’ve got three different learning styles addressed there.*

[Health knowledge expert, P2]

The MOOC incorporated a mix of media across all modules, including graphics, infographics, videos, transcripts, and hyperlinks. It included 30 videos featuring subject area experts (eg, epidemiologists, neurologists, cardiologists, paramedics, and dietitians), 28 videos featuring individuals with lived experience of stroke and carers, and 2 hand-drawn animated videos. Videos were concise, with an average duration of 1 minute and 47 seconds. While most suggestions were incorporated into the MOOC, the absence of multilingual options or features limited its accessibility to people from culturally and linguistically diverse (CALD) backgrounds. Notably, this was already identified as the next step in the broader project after evidence is established for this MOOC.

## Discussion

### Principal Findings

We describe the co-design of a MOOC, including an evaluation of the co-design process, aimed at improving knowledge of stroke and supporting the identification and management of stroke risk factors in the community. To the best of our knowledge, the Preventing Stroke MOOC is the first MOOC worldwide for primary prevention of stroke. The process involved active multiphase co-design with health knowledge experts, community members, and the design team. The co-design process identified topics to support a comprehensive understanding of stroke, along with delivery modes and engagement features to ensure accessibility and relevance to the intended audience. The Preventing Stroke MOOC, which included 7 core modules and 9 mini-modules, was viewed positively by participants and perceived as a useful tool for improving stroke knowledge. Through evaluating the co-design process of the final MOOC features against the original co-design recommendations, we identified gaps to inform future refinements to the MOOC.

Previous research identifies low engagement and completion as major challenges for MOOCs [36]. These challenges are often attributed to limited consideration of learners’ individual needs in MOOC design, thus limiting its effectiveness [37]. Claflin et al [19] noted that engaging stakeholders early improves the quality of a MOOC and fosters success. We used co-design to develop the Preventing Stroke MOOC, involving diverse participant groups throughout the process. This ensured that the course was relevant to the needs of the intended audience and fit for purpose.

Involving participants ensured that desired features of the MOOC were identified and tailored to diverse learning needs. Participants suggested including interactive activities and multimodal content delivery to accommodate different learning preferences. These suggestions highlight the importance of designing educational content that is accessible to learners with diverse educational needs. This aligns with existing studies on MOOC engagement, which emphasize the use of interactive features and multimodal content to support motivation, retention, and completion across literacy levels [38,39]. Our findings confirm evidence from the MOOC literature regarding learners’ preferences, demonstrating that interactive activities and personalized learning are desired features of MOOCs [40,41].

Research using co-design methods in stroke has primarily focused on secondary prevention and rehabilitation, making our study a unique contribution [42]. Previous studies have mostly used co-design to develop interventions for individual cardiovascular risk factors such as physical inactivity and diet [18,43]. Our inclusion of all major risk factors for stroke in the MOOC is supported by evidence that interventions targeting multiple risk factors have a greater impact on reducing cardiovascular risk in both general and at-risk populations [44].

The evaluation of the co-design process demonstrated that most participants’ suggestions from the co-design phase were reflected in the final MOOC design. Although co-design methodologies are increasingly used to develop health interventions “with” and “for” end users, the process is rarely evaluated in terms of whether the end product reflected the needs and suggestions of co-design participants [21]. Retrospective evaluation is essential to understand how the co-design process shaped the end product, why some suggestions were not addressed, and how future co-design could be improved [21]. Our evaluation of the co-design process addresses a recognized gap in the literature, as previous studies have relied on user feedback or researchers’ reflections on the design process [21]. By providing a practical and transparent approach for retrospectively examining how participants’ contributions are reflected in the final intervention design, this study contributes to the growing body of literature on evaluating the co-design process in health intervention development.

Evidence suggests that many MOOCs lack coherent instructional design principles, such as learning objectives and activities, which limit their effectiveness in achieving intended learning outcomes [37]. Findings from our evaluation of the co-design process informed refinements to the MOOC design prior to its public release.

Suggestions from participants that were not reflected in the MOOC included consistent learning objectives across mini-modules, an aphasia-friendly format, and multilingual options. Inconsistencies in learning objectives may affect learners’ clarity about what to expect when completing these modules. Learning objectives were mostly absent from the mini-modules, which focused on specific individual risk factors and action plans, and therefore may not have required the same structured learning objectives as the core modules, which covered multiple topics. However, inconsistencies should be addressed to ensure coherence.

Co-design participants emphasized using plain language and content aligned with health literacy principles to support comprehension across different literacy levels. Readability scores varied across individual modules, with 7 modules (of 16) scoring below 60, indicating fairly difficult readability for the general population (grade 10‐12, aged 15 to 18 years) [45]. Efforts were taken to simplify language by engaging a professional writer who adapted the draft content of the MOOC into accessible language. Through retrospective evaluation of the co-design process, conducted separately from the design and implementation process, the analysis identified a mismatch between participants’ emphasis on plain language and the readability of the final MOOC content. One contributing factor may be the inclusion of medical terminology, which is often multisyllabic and difficult to further simplify without compromising accuracy. To mitigate this, the MOOC included a glossary section and features such as hover-over definitions for technical terms, thereby providing simplified definitions to support understanding of complex terminology.

Modules with low readability scores may present challenges for learners with low literacy in terms of engaging with and understanding the content [45]. This highlights important accessibility considerations for groups with lower education levels, lower socioeconomic status, and CALD populations, who are also at greater risk of stroke and may benefit most from the MOOC [46]. An important consideration in disseminating health information is to ensure that it is accessible to the broader community [47]. This is particularly relevant in primary prevention, where individuals often have limited motivation to engage with health information [48]. As the purpose of the Preventing Stroke MOOC is to educate the general community with evidence-based and accessible information, it is important to ensure that the content accommodates learners with diverse literacy levels.

Aphasia-friendly formatting was not included in the MOOC. Language deficits such as aphasia are common among stroke survivors but not among the general population [49], so this functionality was of lower priority. However, we agree that such adaptation would be necessary for digital platforms aimed at secondary stroke prevention [49]. Multilingual options within the MOOC were not incorporated, mostly due to time and cost constraints. However, it will be considered for the future to make the platform accessible to people who speak languages other than English.

### Strengths and Limitations

An important strength of the study is the involvement of stakeholders in co-designing the MOOC throughout the design process. This approach aligns with person-based methods and extends beyond consultation and acceptability of the intervention [50]. Furthermore, the co-design focus groups were conducted separately for the health knowledge experts and community member groups, thereby helping to mitigate power imbalances. By adopting a comprehensive approach to reporting the co-design process across 3 phases, we provide valuable insights into planning and designing a MOOC for stroke prevention, an approach that could also be adopted to co-design MOOCs across various complex health interventions.

Our study has some limitations to consider. Our co-design participant groups did not broadly represent the Australian population, and insights from CALD groups were not captured as this was beyond the scope of this initial development. Given that Australia is a multicultural country, our future work will focus on making the Preventing Stroke MOOC accessible to various CALD groups with limited English proficiency and literacy levels.

Community member participants in our study primarily comprised stroke survivors. While we attempted to recruit more broadly from the general community, participation from individuals without a history of stroke was limited, with only 25% (3/12) of community participants having no personal history of stroke. This reflects a broader challenge in engaging individuals at risk of stroke, who often have limited awareness or risk perception, including optimism bias, and may therefore be less likely to engage in prevention initiatives [51-53].

To ensure the relevance of the MOOC content to primary stroke prevention, co-design discussions were framed around primary prevention. Participants with lived experience of stroke contributed insights into missed opportunities for prevention, including gaps in awareness of risk factors, recognition of stroke symptoms, and early preventive action [53,54]. This was also informed by several of these people being volunteers who delivered stroke prevention talks in the community, giving them additional insights about knowledge and awareness among people without stroke. These insights informed the design of the content that aims to improve stroke knowledge, reduce false reassurance among individuals who may underestimate their risk, and encourage engagement with preventive behaviors. However, we acknowledge that these perspectives may differ from those of individuals without direct experience of stroke and should be considered when interpreting the findings. Participation was also limited to a relatively small, self-selected group, which may have included individuals who were more engaged or health-aware than the broader community. For example, community member participants without a history of stroke were carers or individuals interested in stroke prevention who regularly volunteered in community stroke prevention programs and therefore may have had greater awareness of stroke and interest in prevention. Additional strategies may be needed to better engage individuals at risk of stroke in co-design in the future.

In this study, sample adequacy was considered using the concept of information power rather than data saturation [55]. Information power emphasizes the relevance and richness of information contributed in relation to the study aim when considering sample adequacy [55]. Consistent with this approach, the focus was on obtaining rich and relevant insights throughout the co-design process.

We acknowledge the potential limitations of a single researcher conducting the coding, which meant that interrater reliability could not be assessed. While the use of design elements adapted from the ADDIE model in the final stage provided a structured and consistent approach for organizing and comparing findings to reduce variance, we cannot eliminate the possibility of variation in coding. We believe that the primary aim of interpreting and comparing participants’ input with the MOOC design should be considered when interpreting the findings. We also note ongoing debate in qualitative research regarding the role of multiple coders, particularly in interpretive approaches where emphasis is placed on depth of understanding [35]. We mitigated risks associated with one coder by having iterative discussions within the research team to review and refine interpretations to enhance the trustworthiness of the results [29,33].

There is currently no established guidance on retrospectively evaluating the co-design process of an end product. Future research is warranted to establish guidelines for rigorous retrospective evaluation of the co-design process to ensure the end product reflects the needs, priorities, and lived experience of all stakeholders.

### Conclusion

Through a comprehensive co-design process, we have developed a Preventing Stroke MOOC based on suggestions from key stakeholders. It offers great potential to educate the community about stroke and support the recognition and management of stroke risk factors. Overall, this study provides important insights into the educational information, design features, and delivery approaches considered important by stakeholders for primary stroke prevention. In our future work, we will assess the acceptability, engagement, and effectiveness of the Preventing Stroke MOOC in improving stroke knowledge and the uptake of healthy behaviors in a fully powered randomized controlled trial before its public release in 2026.

## Supplementary material

10.2196/89358 Multimedia Appendix 1 Pilot module user testing focus group discussion guide.

10.2196/89358 Multimedia Appendix 2 Co-design phase focus group discussion topics relevant to the MOOC design.

10.2196/89358 Multimedia Appendix 3 Preventing Stroke MOOC design features.

10.2196/89358 Multimedia Appendix 4 Example of analytic process.

10.2196/89358 Checklist 1 COREQ checklist.

## Abbreviations

ADDIE: Analysis, Design, Development, Implementation, and Evaluation
CALD: culturally and linguistically diverse
COREQ: Consolidated Criteria for Reporting Qualitative Research
CRG: community reference group
FAST: face, arms, speech, and time
MCQ: multiple-choice question
MIMR: Menzies Institute for Medical Research
MOOC: massive open online course
UTAS: University of Tasmania
