# Supplementary material for: A Massive Open Online Course for Primary Prevention of Stroke: Evaluation of Co-Design Process
Source: JMIR Cardio. 2026 Oct 20;10:e89358. doi: 10.2196/89358 (PMC13684351; doi:10.2196/89358)
Supplement: Supplementary file 1 [file cardio-v10-e89358-s004.pdf]

Multimedia Appendix 5

Example of analytic process

Raw transcript

"The fact that blood pressure has such a big relationship with stroke."

↓

Meaning unit

Emphasis on the strong relationship between blood pressure and stroke

↓

Interpreted suggestion

Highlight the role of blood pressure as a key modifiable risk factor for stroke

↓

MOOC feature (comparison)

Content includes a dedicated mini-module focused on blood pressure as a key biomedical risk factor for stroke

↓

ADDIE adapted design element (organization)

Content

↓

Conclusion

Suggestions reflected
