# Supplementary material for: A Massive Open Online Course for Primary Prevention of Stroke: Evaluation of Co-Design Process
Source: JMIR Cardio. 2026 Oct 20;10:e89358. doi: 10.2196/89358 (PMC13684351; doi:10.2196/89358)
Supplement: Supplementary file 2 [file cardio-v10-e89358-s001.docx]

**Multimedia Appendix 2**

Pilot module user testing discussion guide:

| Before focus groups (while engaging with the pilot module) | 1. Which topics within the module did you find most useful and/or learn most from? 2. Which aspects did you find most engaging? (e.g. videos, activities, discussion board) 3. Did you have any technical difficulties using the course? (e.g. enrolment and navigation through the course, links to additional material, need and availability of technical support) 4. What could be improved in the module? |
| --- | --- |
| During the focus groups | 1. What type of device did you use to complete the module? 2. About how long did it take to complete the module? 3. Did the time taken to complete the module feel about right? Or was it too long, or too short? 4. Do you think the module is sufficiently interesting/engaging that people will continue with the course and complete additional modules? 5. What did you think of the image on the landing page? 6. Is there anything else you’d like to say about testing the module? |
