# Supplementary material for: A Massive Open Online Course for Primary Prevention of Stroke: Evaluation of Co-Design Process
Source: JMIR Cardio. 2026 Oct 20;10:e89358. doi: 10.2196/89358 (PMC13684351; doi:10.2196/89358)
Supplement: Supplementary file 3 [file cardio-v10-e89358-s002.docx]

**Multimedia Appendix 3**

Co-design phase focus group discussion topics relevant to the MOOC design.

Purvis T, Burns C, Barker S, Kilkenny MF, Gall SL, Farmer C, et al. Co‐Designing a Digital Stroke Prevention Platform: Leveraging Lived Experience and Expert Advice. Health Expectations. 2025;28:e70293.

| **Focus group** | **Objectives and prompt questions** |
| --- | --- |
| 1 | Objectives   - Introduce the project, research staff and participants including ice‐breaking activities - Identify what the community would like to learn about preventing stroke - Determine what would make a stroke prevention program useful - Discuss topics the digital platform should cover   Prompt questions   - What do you think community members want to learn about preventing stroke? - What would make a program about preventing stroke useful to the community? - Which topics are most important to cover? |
| 2 | Objectives   - Summary from prior focus group +/− questions - Identify stroke statistics that would motivate someone to participate in the digital platform. - Determine the preferred format and presentation of stroke statistics (e.g. questions or statements, global or Australian, general or age/sex specific, different impact types, technical terms if explained, imagery)   Prompt questions   - Why would someone want to participate in the digital platform? - What numbers are important when talking about the impact of stroke? |
| 3 | Objectives   - Summary from prior focus group +/− questions - Identify the aspects of infographics and videos that effectively convey information about stroke (What is stroke? What is the impact of stroke? What are the signs of stroke [FAST]?)   Prompt questions   - What format and features of the presentation style and delivery are preferred/not preferred? - Consider:   - Type of information (e.g. analogies),   - Level of detail (e.g. physiology, anatomy)   - Format type (e.g. infographic vs illustration)   - Colouring   - Text (e.g. written and spoken)   - Credibility of the information   - Video (length, background) |
| 4 | Objectives   - Summary from prior focus group +/− questions - Prioritise common and uncommon risk factors to include in the digital platform - Review the style of messaging and level of detail (using blood pressure as an example)   Prompt questions   - What are the important risk factors to be included in a program about preventing stroke? - What information should be included for each risk factor? For example definition, effects, management. Case example of blood pressure used. |
| 5 | Objectives   - Summary from prior focus group +/− questions - Identify practical tips for making the most of a doctor visit for stroke prevention (getting people to the doctor, making the most of their visit, the most important question to ask, feedback on the Heart Health Check video) - The next steps for enacting change to prevent stroke   Prompt questions   - What would help people get to the doctor? - What actions can people take to get the most of out of their doctor's visit? - What is the most important question to ask you doctor about preventing stroke? - What are the next steps to action to prevent stroke? |
| 6 | Objectives   - Summary from prior focus group +/− questions - Identify factors which may increase participant engagement with the digital platform (with the MOOC modules and text messages discussed separately)   Prompt questions   - What factors may influence engagement with the MOOC? - What factors may influence finishing the MOOC? - What factors may influence people reading the text messages? |
