# Supplementary material for: A Massive Open Online Course for Primary Prevention of Stroke: Evaluation of Co-Design Process
Source: JMIR Cardio. 2026 Oct 20;10:e89358. doi: 10.2196/89358 (PMC13684351; doi:10.2196/89358)
Supplement: Supplementary file 4 [file cardio-v10-e89358-s003.docx]

**Multimedia Appendix 4**

Preventing Stroke Massive Open Online Course design features organized according to design elements adapted from the Analysis, Design, Development, Implementation, and Evaluation model.

| **Module** | **Learning objectives** | **Structure** | **Content** | **Exercise** | **Assessment** | **Media selection** |
| --- | --- | --- | --- | --- | --- | --- |
| Module 1: Introduction | Clearly stated | Linear | Welcome → Overview of modules → Personal health consideration → Menzies Institute for Medical Research and Stroke foundation → Meet the MOOC academic team → Meet people with lived experience → Navigation tips | n/a | n/a | 1 video  1 PDF (video transcript)  12 hyperlinks |
| Module 2: What is stroke | Clearly stated | Linear | Anatomy and function of the brain → Two main types of stroke → Transient ischemic attack → Stroke affects functions of the body → Lived experience stories on stroke types and loss of function → Module summary | Crossword  Layer the different parts of the brain | MCQ | 8 infographics  7 videos  7 PDF (video transcript) |
| Module 3: Stroke numbers | Clearly stated | Linear | How do epidemiologists use stroke numbers? → Where do stroke numbers come from? → Burden of disease → How often does stroke happen? → Who experiences stroke? → Childhood and perinatal stroke → Stroke in adults → What happens after a stroke → Stroke numbers (outcomes) → lived experience of stroke → Further reading → Module summary | True/False question | MCQ | 19 graphics  9 videos  9 PDF (video transcript)  6 hyperlinks |
| Module 4: Signs of stroke | Clearly stated | Linear | Know signs of stroke → Other signs of stroke → Always call an ambulance → Stroke is time critical → Stroke diagnosis → Stroke treatment → Lived experience participants explain what the F.A.S.T. messaging means to people who have had a stroke → Rapid stroke treatment in regional and remote areas → Telestroke → Module summary | Flashcards to name F.A.S.T. acronym | MCQ | 15 graphics  1 infographic  9 videos  9 PDF (video transcript)  12 hyperlinks |
| Module 5: Risk factors | Clearly stated | Modular learning with optional element | What is a risk factor? → Biomedical and behavioral risk factors → Risk factors for stroke → Stroke risk factors we cannot change → Stroke risk factors we can manage or reduce → Uncommon causes of stroke → Do you know your stroke risk? → Module summary → Further readings | 10-question to assess cardiovascular health with feedback  Image choice to select modifiable risk factors for stroke | MCQ | 10 graphics  1 infographic  4 videos  4 PDF (video transcript)  11 hyperlinks |
| Module 5.a: Blood pressure* | Not stated | Linear | What is blood pressure? → Why does high blood pressure cause stroke? → How is blood pressure measured? → How can you treat high blood pressure? → Further reading → Module summary | Not included | MCQ | 2 graphics  1 infographic  4 videos  4 PDF (video transcript)  7 hyperlinks |
| Module 5.b: Cholesterol* | Not stated | Linear | What is cholesterol and what does it do? → Why does high cholesterol cause stroke? → How is cholesterol measured? → What causes high cholesterol? → How can you treat high cholesterol? → Further reading → Module summary | Not included | MCQ | 1 graphic  2 videos  2 PDF (video transcript)  6 hyperlinks |
| Module 5.c: Atrial fibrillation* | Not stated | Linear | What is atrial fibrillation? → Why does atrial fibrillation cause stroke? → What causes atrial fibrillation? → How can you treat atrial fibrillation? → Further reading → Module summary | Not included | MCQ | 5 graphics  1 infographic  3 videos  3 PDF (video transcript)  4 hyperlinks |
| Module 5.d: Overweight & obesity* | Clearly stated | Linear | What are overweight and obesity? → What is Body Mass Index? → Unhealthy body fat distribution → Overweight and obesity is a risk factor for stroke → What causes overweight and obesity? → What can you do to manage overweight and obesity? → Further reading | Not included | MCQ | 2 graphics  2 infographics  2 videos  2 PDF (video transcript)  6 hyperlinks |
| Module 5.e: Blood sugar & diabetes* | Not stated | Linear | What is diabetes? → Type 1 and Type 2 diabetes → Insulin and blood sugar → Prediabetes → Diagnosing diabetes → Why is diabetes a risk factor for stroke? → Managing diabetes to reduce stroke risk → Further reading → Module summary | Not included | MCQ | 8 graphics  2 videos  2 PDF (video transcript)  6 hyperlinks |
| Module 6: Action plan | Clearly stated | Modular learning with optional element | Managing your stroke risk → Know your risk → Live tobacco free → Eat a healthy diet → Be physically active → Get adequate sleep → Further reading → Module summary | Crossword | MCQ | 6 graphics  1 infographic  3 videos  3 PDF (video transcript)  19 hyperlinks |
| Module 6.a: Smoking* | Clearly stated | Linear | Smoking and stroke → Quitting smoking with potential strategies → Three main reasons for smoking → Further reading → Module summary | Not included | MCQ | 1 graphic  3 videos  3 PDF (video transcript)  7 hyperlinks |
| Module 6.b: Diet & alcohol* | Clearly stated | Linear | What to eat? → Why is healthy eating important? → Fruit and vegetables → Wholegrains → Salt → Meal plans → Alcohol consumption → Further reading → Module summary | Not included | MCQ | 5 graphics  6 infographics  4 videos  4 PDF (video transcript)  17 hyperlinks |
| Module 6.c: Exercise* | Clearly stated | Linear | What is physical activity? → How much physical activity should we do? → Why is physical activity important for stroke? → Australian Heart Foundation tips for getting started → Further reading → Module summary | Not included | MCQ | 3 graphics  2 videos  2 PDF (video transcript)  7 hyperlinks |
| Module 6.d: Sleep & wellbeing* | Clearly stated | Linear | Get adequate sleep → Benefits of good sleep → How to get good sleep → Mental health and wellbeing → Further reading → Module summary | Not included | MCQ | 2 graphics  4 videos  4 PDF (video transcript)  9 hyperlinks |
| Module 7: Completion | n/a | Linear with optional element | Congratulations → Summary of modules → Feedback on the MOOC → Discussion forum → Email to get in touch → Certificate of completion | 1 Discussion board | n/a | 10 graphics  1 video  1 PDF (video transcript) |

* *Mini modules* ; MCQ = multiple choice question; n/a = not applicable
