# Supplementary material for: A Massive Open Online Course for Primary Prevention of Stroke: Evaluation of Co-Design Process
Source: JMIR Cardio. 2026 Oct 20;10:e89358. doi: 10.2196/89358 (PMC13684351; doi:10.2196/89358)
Supplement: Supplementary file 5 [file cardio-v10-e89358-s005.docx]

**Multimedia Appendix 1**

**Consolidated criteria for reporting qualitative studies (COREQ): 32-item checklist**

Developed from: Tong A, Sainsbury P, Craig J. Consolidated criteria for reporting qualitative research (COREQ): a 32-item checklist for interviews and focus groups. International Journal for Quality in Health Care. 2007;19(349 – 357)

| **No. Item** | **Guide questions/description** | **Reported on Page #** |
| --- | --- | --- |
| **Domain 1: Research team and reﬂexivity** |  |  |
| *Personal Characteristics* |  |  |
| 1. Interviewer/facilitator | Which author/s conducted the interview or focus group? | 6, 7, 8, 10 |
| 2. Credentials | What were the researcher’s credentials? E.g. PhD, MD | 7, 8 |
| 3. Occupation | What was their occupation at the time of the study? | 7, 8 |
| 4. Gender | Was the researcher male or female? | 7 |
| 5. Experience and training | What experience or training did the researcher have? | 7, 8 |
| *Relationship with participants* |  |  |
| 6. Relationship established | Was a relationship established prior to study commencement? | 6 |
| 7. Participant knowledge of the interviewer | What did the participants know about the researcher? e.g. personal goals, reasons for doing the research | 6, 7, 8 |
| 8. Interviewer characteristics | What characteristics were reported about the interviewer/facilitator? e.g. Bias, assumptions, reasons and interests in the research topic | 6, 7 |

| **Domain 2: study design** |  |  |
| --- | --- | --- |
| *Theoretical framework* |  |  |
| 9. Methodological orientation and Theory | What methodological orientation was stated to underpin the study? e.g. grounded theory, discourse analysis, ethnography, phenomenology, content analysis | 11 |
| *Participant selection* |  |  |
| 10. Sampling | How were participants selected? e.g. purposive, convenience, consecutive, snowball | 6, 8 |
| 11. Method of approach | How were participants approached? e.g. face-to-face, telephone, mail, email | 6, 8 |
| 12. Sample size | How many participants were in the study? | 14, 15, 16, 17 |
| 13. Non-participation | How many people refused to participate or dropped out? Reasons? | 14, 15, 16, 17 |
| *Setting* |  |  |
| 14. Setting of data collection | Where was the data collected? e.g. home, clinic, workplace | 6, 7, 8, 9, 10 |
| 15. Presence of non-participants | Was anyone else present besides the participants and researchers? | 11 |
| 16. Description of sample | What are the important characteristics of the sample? e.g. demographic data, date | 7, 8, 14, 15 |
| *Data collection* |  |  |
| 17. Interview guide | Were questions, prompts, guides provided by the authors? Was it pilot tested? | 8 |
| 18. Repeat interviews | Were repeat interviews carried out? If yes, how many? | In co-design phase, six focus groups were conducted with each group. |
| 19. Audio/visual recording | Did the research use audio or visual recording to collect the data? | 7, 8 |
| 20. Field notes | Were ﬁeld notes made during and/or after the interview or focus group? | 7, 8 |
| 21. Duration | What was the duration of the inter views or focus group? | 14, 15, 17 |
| 22. Data saturation | Was data saturation discussed? | No, data saturation was not a primary aim of this study, as sample size was guided by information power, with participants selected to provide relevant and rich information to address the study aim rather than to achieve thematic saturation. |
| 23. Transcripts returned | Were transcripts returned to participants for comment and/or correction? | No, transcripts were not returned to participants. Key points were summarised during the focus groups to allow participants to clarify and confirm their responses. In addition, a one-page summary of the process and outcomes was shared with all participants at the end of the co-design phase and following the post-design phase user-testing, providing participants with opportunities to review the findings. |
| **Domain 3: analysis and ﬁndings** |  |  |
| *Data analysis* |  |  |
| 24. Number of data coders | How many data coders coded the data? | 11, 12 |
| 25. Description of the coding tree | Did authors provide a description of the coding tree? | 11, 12 |
| 26. Derivation of themes | Were themes identiﬁed in advance or derived from the data? | 11, 12 |
| 27. Software | What software, if applicable, was used to manage the data? | 10, 11, 12 |
| 28. Participant checking | Did participants provide feedback on the ﬁndings? | Yes, participants provided feedback on the MOOC during co-design and user testing. |
| *Reporting* |  |  |
| 29. Quotations presented | Were participant quotations presented to illustrate the themes/ﬁndings? Was each quotation identiﬁed? e.g. participant number | Throughout results |
| 30. Data and ﬁndings consistent | Was there consistency between the data presented and the ﬁndings? | Throughout results |
| 31. Clarity of major themes | Were major themes clearly presented in the ﬁndings? | Throughout results |
| 32. Clarity of minor themes | Is there a description of diverse cases or discussion of minor themes? | Throughout results |
